# Unique CdS@MoS_2_ Core Shell Heterostructure for Efficient Hydrogen Generation Under Natural Sunlight

**DOI:** 10.1038/s41598-019-48532-3

**Published:** 2019-08-19

**Authors:** Sunil R. Kadam, Suresh W. Gosavi, Bharat B. Kale, Norihiro Suzuki, Chiaki Terashima, Akira Fujishima

**Affiliations:** 1 Centre for Advanced Studies in Materials Science, Department of Physics, Savitribai Phule Pune University, (Formerly University of Pune) Ganeshkhind, Pune, 411007 India; 2 Centre for Materials for Electronics Technology (C-MET), Ministry of Electronics and Information Technology (MeitY), Government of India, Panchawati, Off. Pashan Road, Pune, 411008 India; 3 Photocatalysis International Research Center, Research Institute for Science & Technology, Tokyo University of Science, 2641 Yamazaki, Noda, Chiba, 278-8510 Japan

**Keywords:** Photocatalysis, Pollution remediation

## Abstract

The hierarchical nanostructured CdS@MoS_2_ core shell was architectured using template free facile solvothermal technique. More significantly, the typical hexagonal phase of core CdS and shell MoS_2_ has been obtained. Optical study clearly shows the two steps absorption in the visible region having band gap of 2.4 eV for CdS and 1.77 eV for MoS_2_. The FESEM of CdS@MoS_2_ reveals the formation of CdS microsphere (as a core) assemled with 40–50 nm nanoparticles and covered with ultrathin nanosheets of MoS_2_ (Shell) having size 200–300 nm and the 10–20 nm in thickness. The overall size of the core shell structure is around 8 µm. Intially, there is a formation of CdS microsphre due to high affinity of Cd ions with sulfur and further growth of MoS_2_ thin sheets on the surface. Considering band gap ideally in visible region, photocatalytic hydrogen evolution using CdS@MoS_2_ core shell was investigated under natural sunlight. The utmost hydrogen evolution rate achieved for core shell is 416.4 µmole h^−1^ with apparent quantum yield 35.04%. The photocatalytic activity suggest that an intimate interface contact, extended visible light absorption and effective photo generated charge carrier separation contributed to the photocatalytic enhancement of the CdS@MoS_2_ core shell. Additional, the enhanced hole trapping process and effective electrons transfer from CdS to MoS_2_ in CdS@MoS_2_ core shell heterostructures can significantly contribute for photocatalytic activity. Such core shell heterostructure will also have potential in thin film solar cell and other microelectronic devices.

## Introduction

In recent years, The worldwide energy crisis and eco-friendly energy demand call for the generation of advanced photocatalytic materials as one of the major solving strategy^1^. In the past few decades various semiconductor photocatalyst have been demonstrated for conversion of solar energy into electrical and chemical energy due to their unique properties of semiconductor catalyst, such as high extinction coefficient of light absorption, tuneable band gap, and charge carrier multiplication effect^2^. Recently, in order to look for clean reproducible energy, considerable attention has been paid to the abundant available natural solar light harvesting for hydrogen generation using semiconductor as a photocatalyst^3^. The transition metal oxide semiconductor catalyst such as ZnO and TiO_2_ reported for best photocatalytic activity. Along with ZnO and TiO_2_ some other oxide catalyst such as SnO_2_^4^, Ta_2_O_5_^5^, Nb_2_O_5_^6^, Co_3_O_4_^7^, WO_3_^8^, V_2_O_5_^9^, MoO_3_^10^, Bi_2_MoO_4_^11^, and CdMoO_4_ also reported^12^. But these materials have wide band gap (>3.0 eV) and show good activity only under UV light which is only 5% of the solar spectrum and did not show good photocatalytic activity in the visible light^13^. Furthermore, there are attempts on development of anion doped metal oxide nanomaterials for visible light active photocatalyst^14^. However, the stability of these materials limits for its commercial use as an active photocatalyst. Hence, researchers are focused their research on the development of stable and efficient visible light active photocatalyst for H_2_ generation^13^.

The transition metal chalcogenide semiconductor catalyst such as CdS reported as one of the promising semiconductor catalyst for hydrogen generation due to its narrow band gap 2.4 eV, proper valence band position and excellent stability^15,16^. The conduction band edge of CdS is more negative than the reduction potential of H^+^/H_2_, making it more suitable for the H_2_ generation^17,18^. There are reports on the synthesis of CdS/MoS_2_ composites via various approaches, which shows the good photocatalytic activity as compared to pristine CdS^19^. However, there is still scope to improve photocatalytic activity of CdS toward water splitting by solving the photo corrosion and faster charge recombination problem^14,20^. Therefore, researcher focused much attention mainly on fabricating nanostructure heterojunction to reduce surface recombination, enhanced visible light absorption and modifying surface defects in order to improve the water splitting activity^21,22^. Each semiconductor in the heterostructures could be properly chosen and tailored independently to complete the designed functionality. Recently, two dimensional layered molybdenum disulfide (MoS_2_) has attracted a great deal of attention in the field of photocatalysis and energy applications, due to its excellent photocatalytic performance and cycling stability^23^. Also the MoS_2_ and CdS have same hexagonal crystal structure, which ensures the intimate heterojunction formation. Hinnemann B. *et al*. from density functional theory calculation, revealed that free energy of atomic hydrogen bonding to MoS_2_ is zero, which is very close to Pt and makes it promising catalyst for hydrogen generation^24^. The experimental and computational results disclosed that the edges of ultrathin MoS_2_ nanosheets act as an active sites for the photocatalytic H_2_ generation^25^. Yan *et al*. demonstrated the CdS/MoS_2_ core shell nano rod synthesis for efficient hydrogen production by a facile chemical deposition method^19^.

Herein, CdS@MoS_2_ core shell fabrication was demonstrated by simple solvothermal route. The hierarchical heterostructures of few layered ultrathin nanosheets of MoS_2_ uniformly grown on the surface of CdS microsphere is demonstrated. The as-prepared CdS@MoS_2_ core shell heterostructures show much higher photocatalytic activity and better stability toward water splitting compared with pristine CdS and MoS_2_ under natural solar light. Our superior photocatalytic hydrogen generation results suggest CdS@MoS_2_ core shell is promising cost effective photocatalyst for the clean energy fuel.

## Experimental Methods

### Material synthesis

All chemicals were purchased from Fisher Scientific with (Purity 99%). All the chemicals were AR grade and used without any further purification. Cadmium nitrate (4 mmol) and ammonium heptamolybdate tetra hydrate (4 mmol) dissolved in 50 ml methanol separately each. Drop by drop addition of nitric acid in ammonium molybdates solution with constant stirring for 10 minutes to see the clear solution. Followed by drop wise addition of dissolved cadmium nitrate solution into ammonium molybdate solution with constant stirring. After complete addition, it was allow to stir for 15 more minutes with drop wise addition of dissolved thiourea, followed by stirring for 15 more minutes. The solution was then packed in Teflon coated hydrothermal reactor and kept for 48 hours at 150 °C. After completion of reaction, reactor was allowed to cool down to room temperature naturally, followed by washing of the product with distilled water and filtered using 0.41 whatman filter paper. Further, products were washed using AR grade ethanol for several times and then kept for drying at 80 °C for 4 hours in heating oven. The dried powder catalyst again annealed in the tubular furnace at 400 °C for 3 hours in inert atmosphere. After completion of the annealing reaction, allow to cool the product naturally and used for further characterization and photocatalytic study.

### Photocatalytic hydrogen generation via water splitting

The photocatalytic water splitting was carried out by using 100 ml double distilled water in 250 ml quartz reactor containing 0.1 g CdS@MoS_2_ core shell catalyst. The 0.25 M Na_2_S and 0.35 M Na_2_SO_3_ solutions were used as sacrificial reagents. Argon gas was purged through this reaction mixture in order to remove the dissolved gases. The reactor has septum arrangement to collect the evolved gas through the gas tight syringe for analysis using gas chromatography. The all assembly was arranged in an open natural solar light. The quartz reactor has arrangement for water circulation in order to absorb the IR radiation which minimizes the overheating effect. As soon as the reactor was exposed to natural sunlight, the photocatalytic splitting of water was started and generated H_2_ collected in a head space of the reactor. The amount of H_2_ evolved and its purity was analyzed by gas chromatograph with time. For comparative study the Photocatalytic activity for H_2_ generation carried out using Xe-lamp light source (LOT ORIEL GRUPPE, EUROPA, LSH302) of intensity 300 W. The apparent quantum efficiency of the hydrogen generated was calculated using the following equation.

$${\rm{AQE}}\,( \% )=\frac{{\rm{Number}}\,{\rm{of}}\,{{\rm{H}}}_{2}\,{\rm{molecule}}\,{\rm{evolved}}\,\times \,2}{{\rm{Number}}\,{\rm{of}}\,{\rm{Incident}}\,{\rm{photon}}}\times 100$$

## Results and Discussion

**Figure 1:**
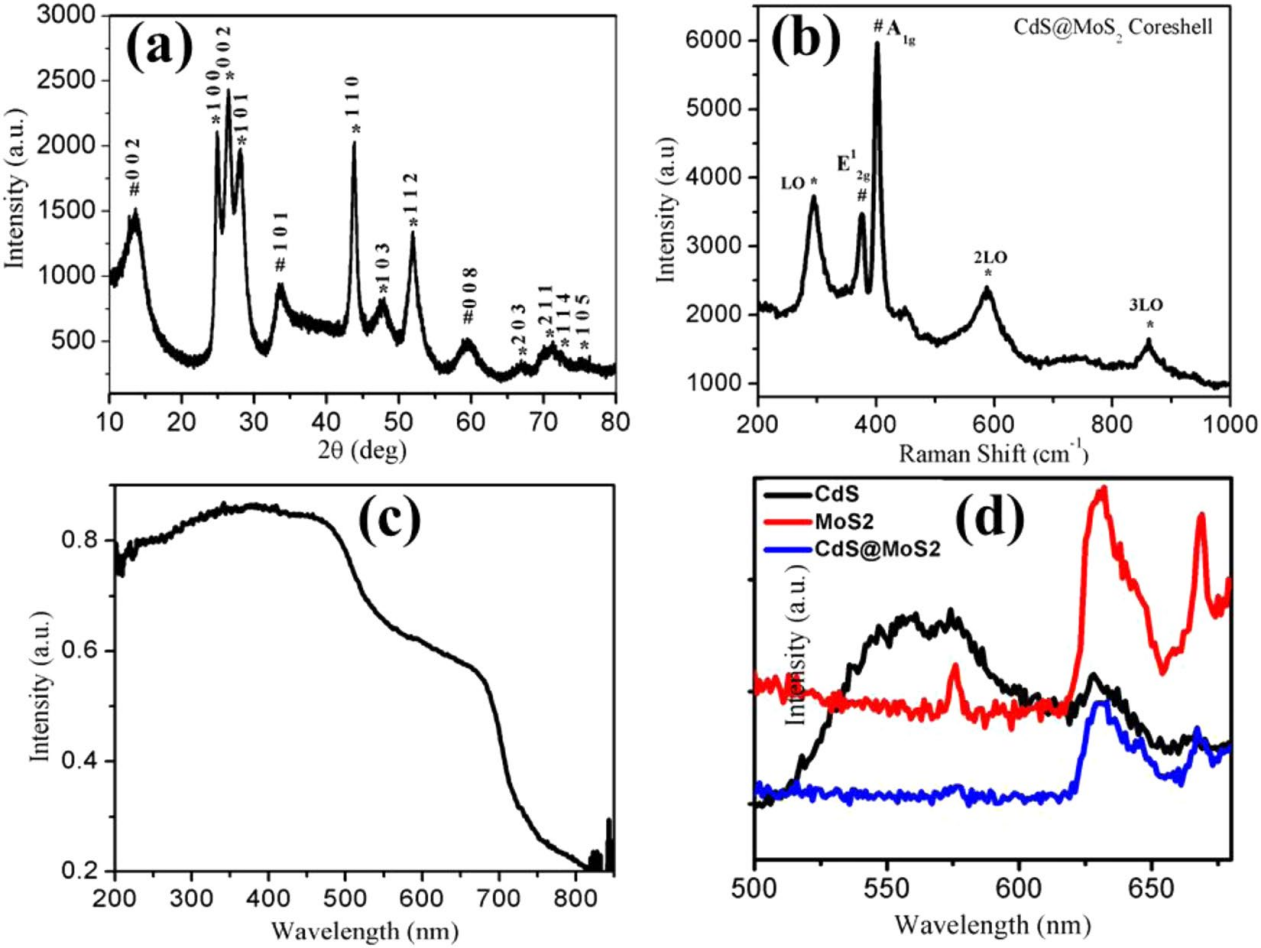
(**a**) XRD of CdS@MoS_2_ core shell. MoS_2_ and CdS peaks are indicated by # and *, respectively. (**b**) Raman spectrum of CdS@MoS_2_ core shell (**c**) UV-Visible absorption spectrum of CdS@MoS_2_ core shell (**d**) Photoluminescence spectrum of CdS@MoS_2_ core shell material along with reference, spectrum of CdS and MoS_2_.

The X-Ray Diffraction technique is used to recognize the crystal structure and phase purity of the samples and results as shown in Fig. 1a. The XRD results confirm the formation of typical hexagonal phase of MoS_2_. The peaks observed at 2θ = 13.9, 33.0 and 58.7 corresponding to the (002), (101) and (008) facets of hexagonal MoS_2_, respectively and matches well with the previous reports of JCPDS card No. 01-075-1539^14^. From the XRD results, it is revealed that MoS_2_ prior growth in crystallinity is at the (002) facet. The XRD (Fig. 1a) also shows the characteristics peaks of hexagonal CdS structure at 2θ = 23.1, 24.9, 26.5, 43.8, 47.8, 51.9, 66.8, 70.9, 72.4 and 75.5 which matches well with the previous reports having JCPDS card No. 01-077-2306. The Fig. 1a clearly reveals that CdS and MoS_2_ are having hexagonal phase. In the XRD (Fig. 1a), MoS_2_ peaks are indicated by #, whereas the CdS peaks are indicated by *.

The Raman measurements were conducted using laser wavelength of 532 nm with laser power 10 mW with a laser spot size 1 μm and the results were shown in Fig. 1b. The broad peak at ~300, 595 and 870 cm^−1^ are assigned to fundamental optical phonon mode (LO) with the first overtone mode (2LO), and second overtone (3LO) of CdS, respectively^14,26^. Raman spectroscopy is very powerful refined technique, used to access phases present and number of layers of MoS_2_ material in terms of the position and frequency difference of two characteristic vibrational modes, E^1^_2g_ and A_1g_^27^. The CdS@MoS_2_ core shell structure shows two peaks centered at 378 and 401 cm^−1^ are assigned as an E^1^_2g_ and A_1g_ vibration mode of MoS_2_. The E^1^_2g_ mode is attributed to the in-plane vibration of Mo and S atoms, while the A_1g_ mode is related to the out-of-plane vibration of S atoms^28^. The frequency difference between the E^1^_2g_ and A_1g_ peak of MoS_2_ measured to be 23 cm^−1^. This value is smaller than that of the bulk MoS_2_, indicating presence of few layered shell of MoS_2_ nanosheets on CdS core^29^. Dravid et. al. well discussed that Raman spectroscopy has also been utilized to investigate the lattice strain and van der waals interaction at the interface of the 2D MoS_2_ nanosheet^28^. The in-plane Raman E^1^_2g_ and A_1g_ mode, is sensitive to the built-in strain of 2D MoS_2_ and the reflection of interlayer van der Waals interactions, respectively^28^. Thus, it is reasonable to predict that the shift of E^1^_2g_ and A_1g_ modes of the MoS_2_ in CdS@MoS_2_ core shell structures towards the lower frequency side attributed to the effect of lattice strain due to the curving of MoS_2_ shell^30,31^.

As the photo absorption properties play a crucial role in determining the photocatalytic activity, UV-visible absorption spectrum of synthesized CdS@MoS_2_ core shell material was recorded, and the result was depicted in the Fig. 1c. The UV-visible absorption spectrum clearly shows the two steps absorption in the visible region. The first absorption peak observed at 510 nm having band gap 2.4 eV is attributed to CdS^32^, whereas the second absorption peak observed at 700 nm having band gap 1.77 eV is attributed to MoS_2_^14,33^. The band gap of the material was also confirmed from tauc plot as shown in Fig. S1 (Supporting Information). The two absorption peak observed in UV-visible absorption study confirms the presence of both CdS and MoS_2_. The sample absorbing more photons with high energy will produce more electron and hole with a high reduction ability to reduce the water molecules into hydrogen. Thus, the sample may exhibit a high hydrogen evolution rate.

Photoluminescence (PL) investigations are useful for the analysis of migration, transfer, and recombination processes of photo induced electron–hole pairs in a semiconductor material^13^. Photoluminescence measurement for CdS@MoS_2_ core shell was recorded at room temperature using excitation wavelength 350 nm and results were shown in Fig. 1d. The CdS sample shows the broad emission peak centered at 560 nm attributed to the energy transition corresponding to the band gap energy. In case of MoS_2_ and CdS@MoS_2_ the two emission peaks are observed at 630 and 670 nm corresponds to band edge transition and defects created at the interface of the core shell and its matches well with the previous reports^34–36^. The more PL intensity for pristine MoS_2_ and CdS sample reveals the faster electron and hole pair recombination. Whereas, the significant decline in PL intensity of CdS@MoS_2_ core shell is due to the efficient photo-carrier separation by band bending (energetic gradient) at hetero-structure interfaces. The CdS@MoS_2_ Core shell structure facilitates the separation of photo induced electron-hole pairs due to the formation of the hetero junction between the CdS and MoS_2_ and make it available for the photocatalytic activity. Here, we have optimized the structure of the core-shell to achieve the optimum performance.

**Figure 2:**
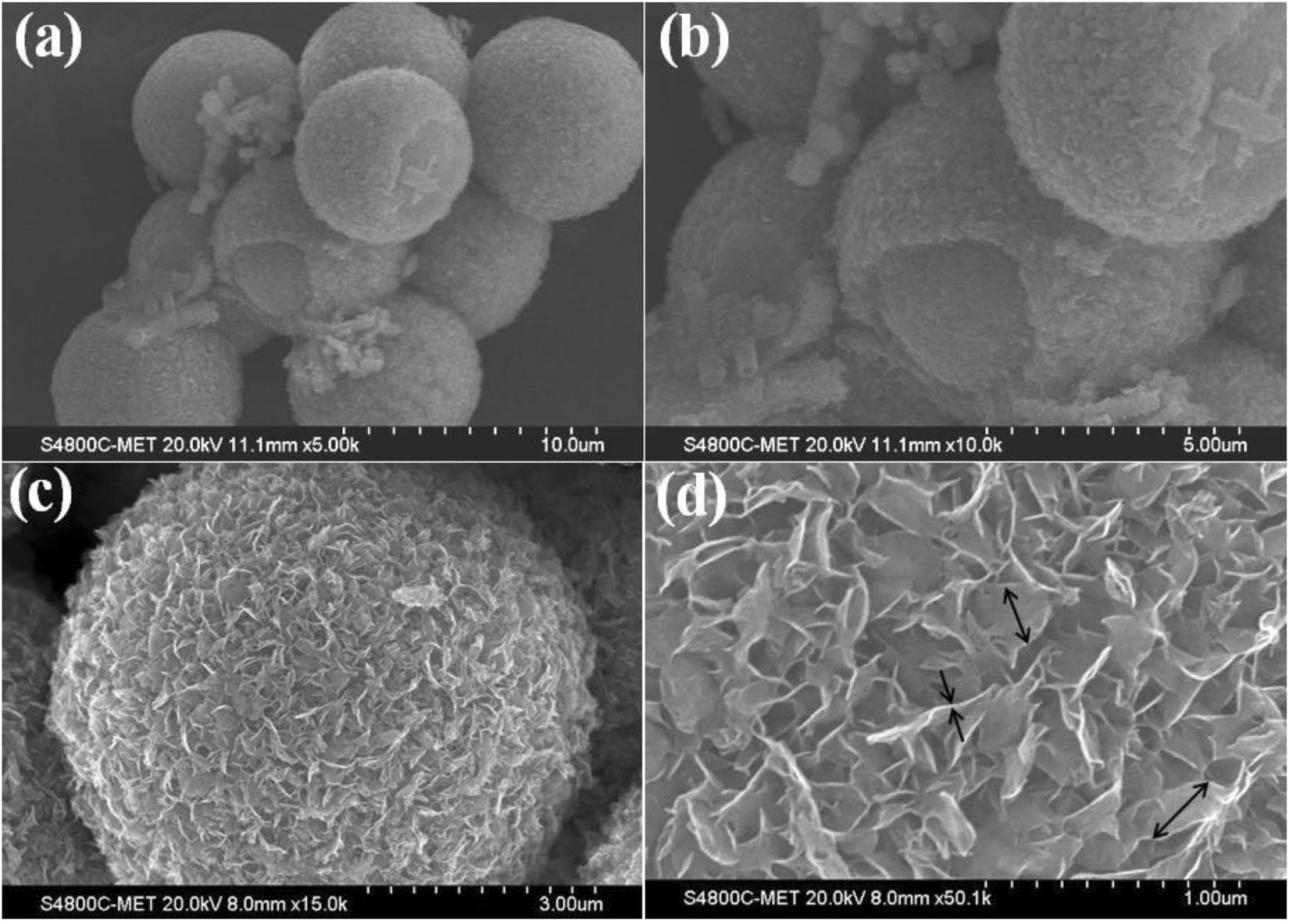
(**a**–**d**) FESEM images of CdS@MoS_2_ core shell with various magnifications.

The surface morphology of the synthesized CdS@MoS_2_ core shell was investigated by FESEM shown in Fig. 2a–d. From FESEM images, it is clearly observed that the formation of the inner core and outer shell like morphology. The average size of the CdS@MoS_2_ core shell was found to be around ~8 µm as shown in Fig. 2a. It is observed that the inner core CdS size is measured to be ~7 µm and CdS core is made from nano particles. Whereas, the thickness of the MoS_2_ shell is observed to be nearly 500 nm in size as observed in Fig. 2a,b. In Fig. 2c,d, it is clearly observed that the outer shell MoS_2_ is composed of small ultrathin nanosheets of size 200–300 nm and average measured thickness is around 10–20 nm as shown in Fig. 2d. It is noteworthy that such unique CdS@MoS_2_ core shell structure using solvothermal process is hitherto unattempted.

**Figure 3:**
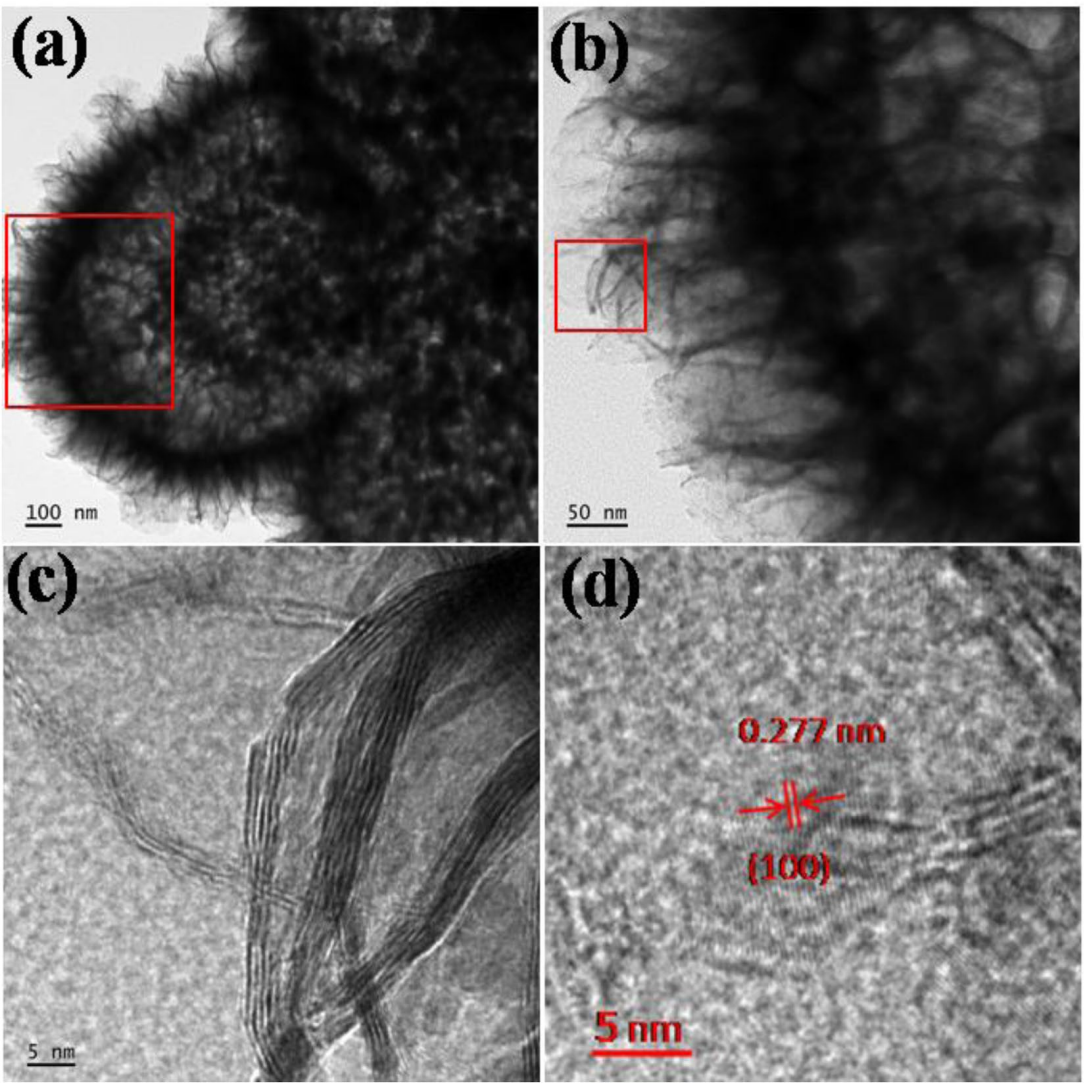
(**a**,**b**) FE-TEM images of CdS@MoS_2_ core shell and (**c**,**d**) High resolution image.

Field emission transmission microscopy (FETEM) was used to further analyse the crystallinity, morphology, size of the particle and ultrathin nano sheets in the CdS@MoS_2_ core shell sample. In a typical TEM image shown in Fig. 3a, it is observed the presence of inner core and outer shell of the CdS@MoS_2_ core shell material. The Fig. 3a clearly reveals, CdS core is composed of particles of 40–50 nm average size. The space between the core and shell is accompanied by the ultrathin nanosheets. The outer surface of the shell covered by ultrathin nanosheets is just feather like appearance as shown in Fig. 3b. In a higher magnification image (Fig. 3c), it reveals that outer shell of MoS_2_ is composed of few layered nanosheets. The d-spacing measured from the HRTEM image taken at the edge of the sheet is 0.277 nm corresponding to the (100) plane of the hexagonal MoS_2_ phase, which in accordance with the XRD results. The selected area electron diffraction taken at the edge of the nanosheet confirms the polycrystalline nature of the MoS_2_ shell as shown in Supporting Information Fig. S2.

**Figure 4:**
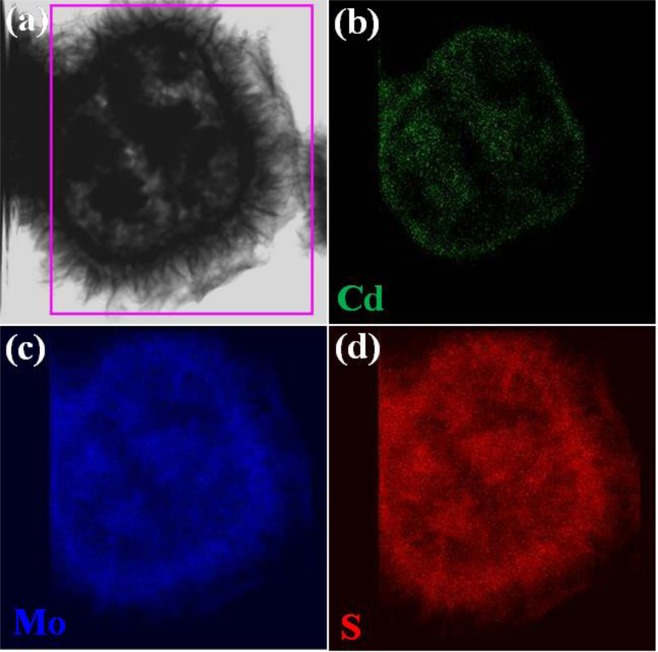
(**a**) TEM image of CdS@MoS_2_ core shell and Corresponding elemental mapping STEM images of (**b**) Cd, (**c**) Mo and (**d**) S element.

The CdS@MoS_2_ core shell material further examined by Scanning transmission electron microscopy (STEM) and elemental results as displayed in Fig. 4. Both Cd and Mo are observed to be uniformly distributed. The Cd signal is appeared in the inner side i.e. core (Fig. 4b). By overlapping the Cd signal, it is found that MoS_2_ seats at outer shell with average thickness of around 500 nm (Fig. 4c), it is consistent of the our FESEM and TEM measurement. The S signal is observed to be uniformly distributed at inner and outer part of the CdS@MoS_2_ core shell material.

**Figure 5:**
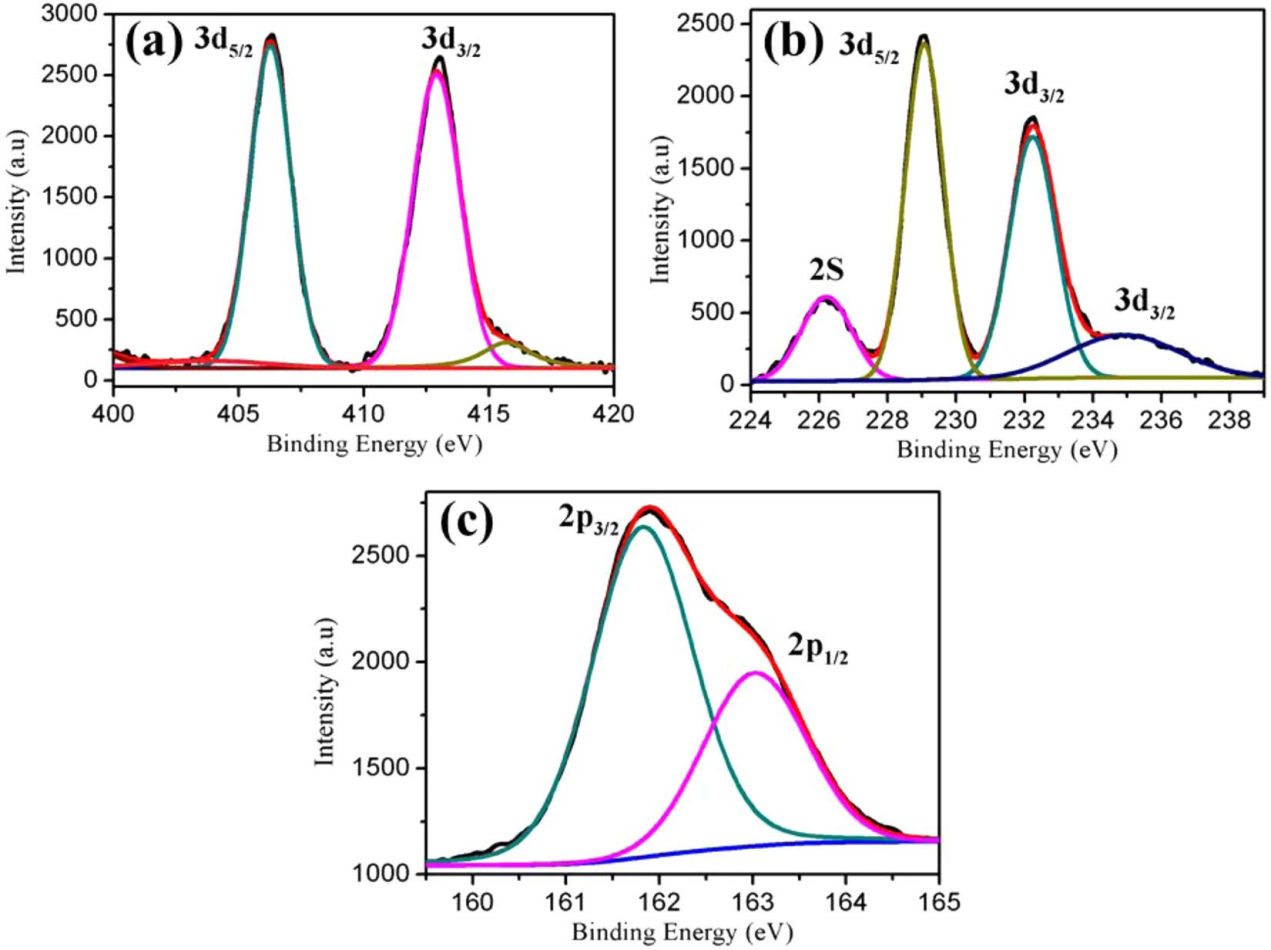
X-ray photoelectron spectra of (**a**) Cd 3d (**b**) Mo 3d and (**c**) S2p of the CdS@MoS_2_ core shell sample.

X-ray photoelectron spectroscopy (XPS) was used to determine the chemical composition and chemical states of the CdS@MoS_2_. Figure 5a shows two peaks at 412.9 and 406.2 eV corresponds to the characteristic binding energies of Cd^2+^ 3d_3/2_ and 3d_5/2_ in CdS, respectively^19^. Figure 5b displays Mo 3d peaks at 229.0 eV and 232.2 eV energy values, corresponding to the 3d_5/2_ and 3d_3/2_ core level peaks. The peak at 226.2 eV matches well with the binding energy of S 2 s in sulfides^22^. The small shoulder peak at 235.5 eV corresponding to 3d_3/2_ of MoO_3_ because of surface oxidation or may be from the starting material^37^. The S 2p peak can be deconvoluted into two peaks at 163.0 eV and 161.8 eV (Fig. 5c), corresponds to the 2p_1/2_ and 2p_3/2_ orbital^19^. These binding energy values are consistent with those reported in previous studies and confirm the expected charge states of Cd^2+^, Mo^4+^ and S^2−^ in the CdS@MoS_2_ core shell. Together with SEM and TEM observation, these results validate the CdS@MoS_2_ core shell.

### Formation and growth mechanism of CdS@MoS_2_ core shell heterostructures

**Figure 6:**
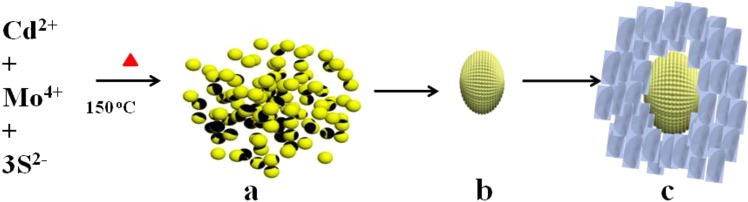
Schematic representation of CdS@MoS_2_ core shell formation. (**a**) CdS nano particles (**b**) CdS microsphere (**c**) CdS@MoS_2_ core shell.

The schematic representation of growth mechanism for CdS@MoS_2_ core shell material is shown in Fig. 6. The CdS@MoS_2_ core shell was prepared solvothermally using cadmium nitrate, ammonium molybdates and thiourea in the methanol medium. During the solvothermal process, the cadmium, ammonium, molybdenum and sulfur ions are formed. Initially, in the hydrothermal reactor Cd^2+^ react with the S^2−^ ion to form the CdS nano particles as per the reaction 1 and shown in Fig. 6a. Initially, the CdS formation preferred due to the high reactivity of the Cd^2+^ ion towards the S^2−^ ion is more than Mo^4+^. Also, the energy require for the CdS formation is lower compare to MoS_2_. The as formed CdS nano particle comes together in order to reduce the surface energy and formation of CdS microsphere takes place (Fig. 6b).

1 $${{\rm{Cd}}}^{2+}+{{\rm{S}}}^{{\rm{2}}-}\to {\rm{CdS}}$$

2 $${{\rm{Mo}}}^{4+}+{{\rm{2S}}}^{{\rm{2}}-}\to {{\rm{MoS}}}_{{\rm{2}}}$$

At solvothermal condition, the Mo^4+^ ions are reacting with the S^2−^ ion in supersaturated solution to form MoS_2_ nuclei. These nuclei further grow by typical crystal growth mechanism and formation of layered ultrathin nanosheets takes place. Due to prolong hydrothermal reaction time, there is a uniform oriental 2D anisotropic growth of these ultrathin nanosheets on the surface of CdS microsphere to form CdS@MoS_2_ core shell (Fig. 6c). This is a very distinctive structure demonstrated in this manuscript, not reported so far in the literature for the CdS@MoS_2_ core shell.

### Photocatalytic activity

The photocatalytic activity of CdS@MoS_2_ core shell material was evaluated by measuring the amount of H_2_ generated via photocatalytic water splitting. The photocatalytic H_2_ generation activity of synthesized material was carried out in presence of solar light. In the present study, we used Na_2_S/Na_2_SO_3_ as a sacrificial reagent. Yang *et al*. earlier discussed the use of Na_2_S/Na_2_SO_3_ as a sacrificial reagent and its detailed mechanism. It seems that presence of Na_2_S/Na_2_SO_3_ decreases the e^−^ and h^+^ pair recombination^38^. The Na_2_S/Na_2_SO_3_ suppresses the evolution of oxygen through the formation of free radicals^39^. As the reactor containing photocatalyst is irradiated with solar light, the e^−^ in conduction band (CB) and h^+^ in valence band (VB) is generated. The photo-generated h^+^ in VB consumed by sulfide S^2−^ and sulfite SO_3_^2−^ enabling the effective separation of charge carriers^40^. While, e^−^ in CB reduces the two H_2_O molecule into one H_2_ gas molecule^12^. The advantages and detailed mechanism of S^2−^/SO_3_^2−^ as scavenger is well discussed in literature by Bahnemann *et al*.^40^. The possible photocatalytic water splitting mechanism is as shown below.

$${\rm{CdS}}@{{\rm{MoS}}}_{{\rm{2}}}\,{\rm{catalyst}}+{\rm{hv}}\to {{\rm{e}}}_{(\mathrm{CB})}^{-}+{{\rm{h}}}_{(\mathrm{VB})}^{+}$$

$${\rm{Reduction}};\,{{\rm{2H}}}_{{\rm{2}}}{\rm{O}}+{{\rm{2e}}}^{-}\to {{\rm{H}}}_{{\rm{2}}}+{{\rm{2OH}}}^{-}$$

**Figure 7:**
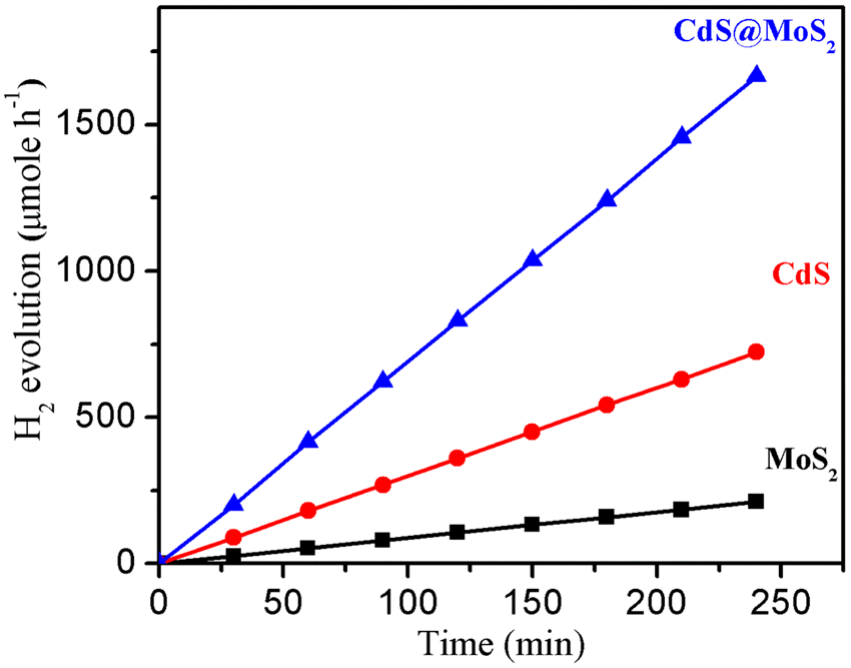
Photocatalytic hydrogen generation activity plot with time for CdS@MoS_2_ core shell along with CdS and MoS_2_.

The results of photocatalytic H_2_ generated in µMole with respect to time for CdS@MoS_2_ core shell material along with CdS and MoS_2_ were shown in Fig. 7 and in Table 1. For comparative study the CdS and MoS_2_ were synthesized at identical condition and tested for the photocatalytic H_2_ generation activity via water splitting reaction under natural solar light. It is clear that CdS@MoS_2_ core shell material shows significantly enhanced H_2_ generation activity as compare with the pristine CdS and MoS_2_. For comparative study, the cumulative H_2_ generation for all the catalysts using solar simulator (xenon lamp) along with H_2_ generation activity under sunlight were tested and obtained results shown in Fig. S3 and Table 1. The highest rate for H_2_ generation under xenon and natural solar light i.e., 231 and 416.4 µMole h^−1^, respectively. The photocatalytic activity results shows less H_2_ generation using xenon lamp as compare to activity obtained in natural solar light. The H_2_ generation achieved in solar light is 416.4, 180.6 and 53.0 µMole h^−1^ for CdS@MoS_2_ core shell, CdS and MoS_2_, respectively. It is important to note that all the photocatalysis experiments reported in this manuscript were carried out at an identical condition. The rate of H_2_ generation for CdS@MoS_2_ is approximately twice and six times more than CdS and MoS_2_, respectively. The utmost hydrogen generation rate achieved for core shell is 4,164 µmole h^−1^ with apparent quantum yield 35.04%, which is much better than other reports for similar materials^22,25,41^. The higher photocatalytic activity obtained for CdS@MoS_2_ core shell is due to heterostructures of CdS@MoS_2_ core shell, which facilitate the photo-carrier separation and prevents carrier recombination. Consequently, the core shell heterostructures can increase large light harvesting efficiency leading to an enhanced photocatalytic activity. Furthermore, the defects created at the interface of CdS@MoS_2_ core shell heterostructures, which ultimately act as a photo-carrier trap and suppresses the recombination significantly. In case of CdS@MoS_2_ core shell the e^−^ and h^+^ generated due to irradiation with solar light at CdS and then they transfer to the ultrathin nanosheets of MoS_2_. The 2D ultrathin nanosheets allows electron to move in the layered structure and make it available for the photocatalytic activity. The ultrathin nanosheets of MoS_2_ have more number of edges, significantly its acts as active sites for the photocatalytic H_2_ generation reaction. From the four hours linear H_2_ generation plot, it is observed there is continuous H_2_ generation in presence of solar light, concludes the good efficiency of the CdS@MoS_2_ core shell catalyst. The stability of the CdS@MoS_2_ catalyst was evaluated by analyzing the XRD of catalyst before and after photocatalysis (Fig. S4, Supporting Information), and the results did not show any significant change in the phase purity of the sample, which reveals the CdS@MoS_2_ core shell catalyst have good stability during the photocatalytic activities. The less hydrogen generation for CdS observed is due to faster carrier recombination and photo corrosion issues in the CdS sample^14^.

**Table 1:** Hydrogen generation via Water splitting.

Sr. No.	Catalyst used	Average H_2_ generated using xenon lamp (µmole)	Average H_2_ generated in Sunlight (µmole)	% Apparent quantum yield
1	CdS	102	180.6	15.19
2	MoS2	29	53.0	4.45
3	CdS@MoS2 Core shell	231	416.4	35.04

This is the only reports which depicts the photocatalytic H_2_ generation under natural sunlight catalyzed by CdS@MoS_2_ core shell catalyst. The activity performance of the catalyst was established by performing reusability study in that catalyst retains its activity after the fifth recycle with negligible decrease, which reveals the reproducibility of the results as shown in Fig. S5 in Supporting Information. We did not found any H_2_ production in the absence of catalyst and in the dark condition (without light). Overall, it is concluded that the hydrogen generation achieved is due to CdS@MoS_2_ core shell catalyst only.

**Figure 8:**
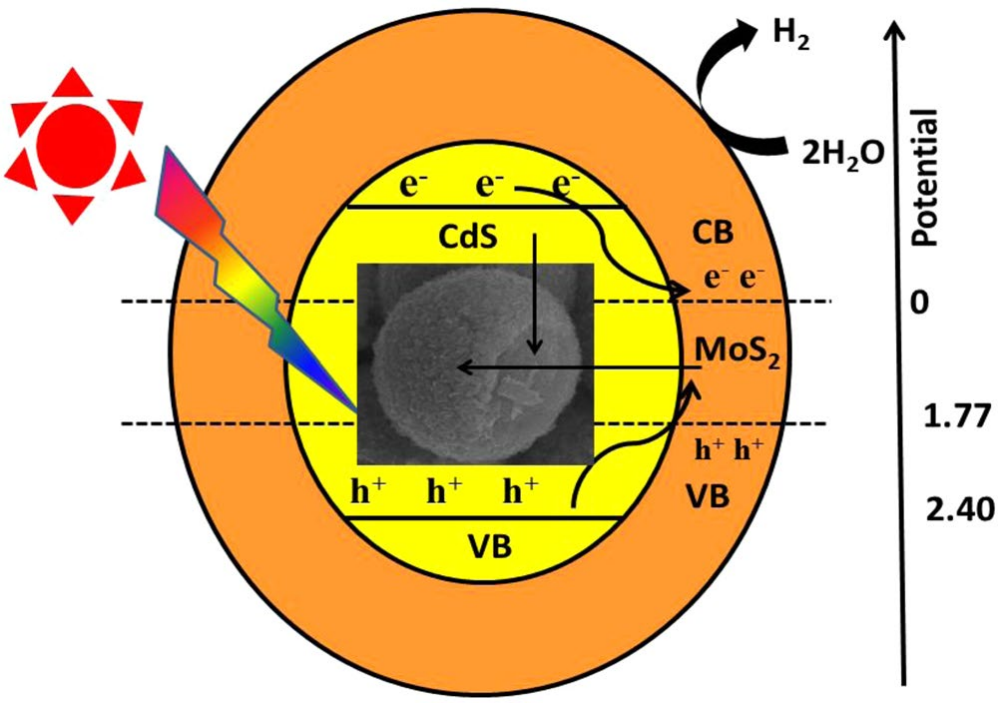
Schematic representation of CdS@MoS_2_ core shell as photocatalyst.

The possible schematic illustration for the photo generated e^−^ and h^+^ pair separation, its transfer process from CdS to MoS_2_ and hydrogen generation mechanism for CdS@MoS_2_ core shell heterostructures is proposed in Fig. 8. The enhanced photocatalytic activity for CdS@MoS_2_ core shell is attributed to the effective electron transfer through the interface formed between the core CdS microsphere and ultrathin nanosheets of MoS_2_ shell as evidenced by the HR-TEM images in Fig. 3, which obviously avert the recombination of the photo generated carriers. The reduction in e^−^ and h^+^ pair recombination of CdS@MoS_2_ core shell is also evidenced by photoluminescence study as shown in Fig. 1d. Under solar light irradiation, the electrons in the valence band (VB) of the CdS core are excited to the conduction band (CB), while the holes are in the VB of CdS. The excited electron in the CB of CdS further transferred into the CB of MoS_2_ due to its lower band position^13^. Whereas the holes from the VB of CdS are trapped at the interface of the CdS@MoS_2_ core shell, which obviously prevents the recombination of photogenerated carriers^10^. The photogenerated holes (h^+^) in the VB of MoS_2_ and at the interface of the CdS@MoS_2_ core shell consumed by sulfide S^2−^ and sulfite SO_3_^2−^. Whereas, the e^−^ from the CB of MoS_2_ take part in the reduction of two H_2_O molecule into one H_2_ molecule. The MoS_2_ nanosheets shell also act as co-catalyst and obviously enhance the transportation of photgenerated electrons and get it available for photocatalytic activity. This fact also supports for the improvement in the photocatalytic activity of the CdS@MoS_2_ core shell.

## Conclusion

In the summary, a facile solvothermal approach for ultrathin MoS_2_ nanosheets shell on CdS microsphere, resulting in unusual CdS@MoS_2_ core shell heterostructures. We observed the photoluminescence quenching phenomenon for CdS@MoS_2_. This may be attributed to the heterostructures effect resulted from the realigned band structure in CdS@MoS_2_. The CdS@MoS_2_ exhibited enhanced photocatalytic hydrogen generation 416.1 µMole h^−1^, which is much better than that of pristine CdS and MoS_2_. The core shell structure of the catalysts played an important key role in the enhancement of H_2_ production. The core shell heterostructures allow faster charge transportation and minimizes the recombination, which subsequently enhance the photocatalytic activity. These finding will open up new avenues for developing low cost heterostructures photo catalyst for water splitting. Our superior photocatalytic hydrogen generation results suggest CdS@MoS_2_ core shell is promising cost effective photocatalyst for the clean energy fuel.

## Supplementary information

Supporting Information
