# Supplementary material for: Unique CdS@MoS2 Core Shell Heterostructure for Efficient Hydrogen Generation Under Natural Sunlight
Source: Sci Rep. 2019 Aug 19;9:12036. doi: 10.1038/s41598-019-48532-3 (PMC6700150; doi:10.1038/s41598-019-48532-3)
Supplement: Supplementary file 1 — Supporting Information [file 41598_2019_48532_MOESM1_ESM.docx]

Unique CdS@MoS_2_ Core Shell Heterostructure for Efficient Hydrogen Generation Under Natural Sunlight

Sunil R. Kadam,^1^ Suresh W. Gosavi,^1*^ Bharat B. Kale^2*^, Norihiro Suzuki^3^, Chiaki Terashima^3^, and Akira Fujishima^3^

*^1^Centrefor Advanced Studies in Materials Science, Department of Physics, SavitribaiPhule Pune University, (Formerly University of Pune) Ganeshkhind, Pune -411007, INDIA*

*^2^Centre for Materials for Electronics Technology (C-MET), Ministry of Electronics and Information Technology (MeitY), Government of India, Panchawati, Off.Pashan Road, Pune –411008, INDIA*

*^3^Photocatalysis International Research Center, Research Institute for Science & Technology, Tokyo University of Science, 2641 Yamazaki, Noda, Chiba 278-8510, JAPAN*

^*^Corresponding author: [swg@physics.unipune.ac.in](mailto:swg@physics.unipune.ac.in), [bbkale@cmet.gov.in](mailto:bbkale@cmet.gov.in)

Material Characterisation

The crystalline nature and phases were investigated using X-ray powder diffraction (XRD) technique (XRD-D8, Advance, Bruker-AXS). Further, Cd, Mo and S content and oxidation state of element were examined using X-ray photoelectron spectroscopy (XPS, Thermo K-ALPHA+Sr.No-KAS2020 Make-M/s Thermo Fisher Scientific, UK). Room temperature Raman spectroscopy were performed using a Renishaw InVia microscope Raman system with a laser wavelength of 532 nm in the back scattering geometry laser power on the sample was 5 mW with a laser spot size 1 μm. The optical properties of the powder samples were studied using an UV-visible-near infrared spectrometer (UV-VIS-NIR, Perkin Elmer Lambda-950). Photoluminescence properties of samples were analyzed by using HORIBA Flurolog 3 spectroflurometer. The morphologies of the as synthesized and annealed sample were characterized by ﬁeld emission scanning electron microscopy (FESEM, Hitachi, S-4800). The FETEM and STEM (JEOL, 2010F instrument) were used to investigate micro structural, crystalline nature and elemental mapping. The samples were prepared by dispersing the powder in ethanol, followed by ultrasonication in an ultrasonic bath for 5 min. and then drop-casting the dispersion on a carbon coated copper grid and by subsequent drying in a vacuum. The purity of the collected gas was analyzed by gas chromatograph (Model Schimadzu GC-14B, MS-5 Å column, TCD, Ar carrier).





Fig. S1: (a) Tauc plot of CdS@MoS_2_ core shell material.





Fig. S2: (a) Selected Area Electron Diffraction pattern MoS_2_ in CdS@MoS_2_ core shell material.





Fig. S3: Photocatalytic hydrogen generation activity under Sunlight and solar simulator for CdS@MoS_2_ core shell along with CdS and MoS_2_.





Fig. S4: XRD of as synthesized CdS@MoS2 core shell sample and its reused sample after photocatalytic hydrogen generation.





Fig. S5: Photocatalytic hydrogen generation stability activity under Sunlight for CdS@MoS_2_ core shell.
